# Retro-inverso follicle-stimulating hormone peptide-mediated polyethylenimine complexes for targeted ovarian cancer gene therapy

**DOI:** 10.1080/10717544.2018.1461956

**Published:** 2018-04-18

**Authors:** Mengyu Zhang, Mingxing Zhang, Jing Wang, Qingqing Cai, Ran Zhao, Yi Yu, Haiyan Tai, Xiaoyan Zhang, Congjian Xu

**Affiliations:** a Obstetrics and Gynecology Hospital, Fudan University, Shanghai, China;; b Department of Gynecology, 411 Military Hospital Affiliated to Changhai Hospital of Shanghai, Shanghai, China;; c Shanghai Key Laboratory of Female Reproductive Endocrine Related Diseases, Shanghai, China

**Keywords:** Ovarian carcinoma, targeted therapy, follicle-stimulating hormone, growth-regulated oncogene α, RNA interference, nanoparticle

## Abstract

**Background:** The development of nanoparticle drug delivery systems with targeted ligands has the potential to increase treatment efficiency in ovarian cancer.

**Methods:** We developed a 21-amino acid peptide, YTRDLVYGDPARPGIQGTGTF (L-FP21) conjugated to polyethylenimine (PEI) and methoxy polyethylene glycol (mPEG) to prepare a nanoparticle drug vehicle to target follicle-stimulating hormone receptor (FSHR) in ovarian cancer. At the same time, we optimized the ligand of the nanoparticle vehicle using D-peptides, which consist of D-amino acids (D-FP21). Nanoparticle vehicles carrying the therapeutic gene plasmid growth-regulated oncogene alpha (pGRO-α) short hairpin RNA (shRNA) (FP21-PEG-PEI/pGRO-α) were prepared for further investigation.

**Results:** Compared with L-FP21, D-FP21 exhibited improved biological stability and higher uptake rate for FSHR-expressing ovarian cancer cells. The cytotoxicity of the L, D-FP21-PEG-PEI/pGRO-α complexes were significantly lower than that of the PEI/pGRO-α complex. The nanoparticle drug with the targeted ligand showed higher transfection efficiencies and improved anti-proliferation effects for ovarian cancer cells than that without the targeted ligand (mPEG-PEI/pGRO-α). Furthermore, an *in vivo* evaluation of an antitumor assay indicated that D-FP21-PEG-PEI/pGRO-α inhibited the growth of tumor spheroids considerably more than L-FP21-PEG-PEI/pGRO-α; their tumor inhibition rates were 58.5% and 33.3%, respectively.

**Conclusions:** D-FP21-PEG-PEI/plasmid DNA is a safe and efficient gene delivery vehicle for ovarian cancer targeted therapy.

## Introduction

Ovarian cancer is the main cause of gynecologic malignancies. Current standard treatment is surgical treatment followed by concurrent or sequential chemotherapy (Xu et al., 2016; He et al., 2016). The problems of nonspecific drug biodistribution leading to strong adverse effects of chemotherapy and drug resistance, giving rise to treatment failure, remain unresolved. The five-year survival rate of patients with ovarian cancer is only 31% (Rauh-Hain et al., 2016). Therefore, there is an urgent need to find an effective method for delivering low drug doses to kill tumor cells in a more concentrated manner.

The development of tumor-targeted therapy holds promise for ovarian cancer therapy (Sapiezynski et al., 2016; Staropoli et al., 2016). A high-specificity antibody or peptide conjugating to the drugs can concentrate more drugs to the tumor microenvironment. Accordingly, the antibody or peptide selected is the key to successful treatment of ovarian tumors (Schiffelers, 2004). Currently, the choice of target sites in ovarian cancer focuses on tumor cell surface molecules such as anti-angiogenesis receptor (Koneru et al., 2015) (vascular endothelial growth factor [VEGF], epidermal growth factor receptor [EGFR/ErbB]), blood markers of ovarian cancer (Li et al., 2015) (mucin 16 [MUC16], human epididymis protein 4 [HE4]), and glycosylated proteases related to suppressing ovarian tumor cell invasion and metastasis (BCL2 interacting killer [BIK]) (Kanhaiya et al., 2017). However, most of them do not move forward to clinical trials because of unsatisfactory treatment effects due to the lack of specificity. Therefore, finding the corresponding target ligands that exhibit high specificity and affinity to tumor cells is an important task in targeted therapy. Follicular maturation is dependent on the development of follicle-stimulating hormone (FSH), and FSH receptor (FSHR) has been found in the ovarian surface epithelium (OSE) and in some ovarian cancer cell lines and tissues; above all, the distribution of FSHR is limited to the reproductive system (Papadimitriou et al., 2016; Perales-Puchalt et al., 2017). This expression pattern makes it possible to use FSHR as the target site against ovarian cancer with high selectivity and specificity. FSH consists of a and h chains, and some FSHR-binding domains have been identified, including amino acids 1 to 15, 33 to 53, 51 to 65, and 81 to 95 of the FSH h chain (Zhang et al., 2009). In our previous study (Zhang et al., 2009), several peptides were designed, and the strongest binding affinity was observed for a 21-amino-acid peptide, YTRDLVYGDPARPGIQGTGTF (FP21), corresponding to the FSH h 33–53 sites. At the same time, nanoparticles were prepared with maleimide-polyethylene glycol (PEG)-polylactide (PLA) and methoxy PEG (mPEG)-PLA, which were synthesized by ring-opening polymerization using the evaporation technique. We found that paclitaxel-loaded nanoparticles conjugated to this peptide could deliver more drugs into FSHR-positive cells. It also facilitated the concentration of drugs to foci to enhance the antitumor efficacy of chemotherapeutic drugs and minimize side effects in unrelated normal organs (Zhang et al., 2009; Fan et al., 2014). An invention patent has been granted for this peptide (patent number: 201610849393.2).

Recently, targeting ligands, especially peptides, have been increasingly used for drug delivery systems. Natural peptides are not stable, especially in blood, because they are easily degraded by the proteases in serum. Their short half-life and instability greatly affect their targeting effect (Tugyi et al., 2005). To overcome this issue, we replaced the L-amino acids of FP21 (L-FP21) with D-amino acids based on the retro-inverso method devised by Chorev and Goodman in 1979 (Li et al., 2015). D-peptides are similar to the parental L-peptides with respect to the side chain structure and thus retain the same bioactivity. Moreover, D-peptides (D-FP21) can theoretically resist protease degradation and exhibit higher stability *in vivo* (Wang et al., 2014).

In therapeutic drug delivery, inorganic nanoparticles have attracted considerable attention because of their good biocompatibility, easy modification, design flexibility, and reduced toxicity (Danhier et al., 2009; Liang et al., 2009; Yin et al., 2014). Polyethylenimine (PEI) is well known in nonviral gene delivery systems because of its high transfection efficiency (Yu et al., 2010) as compared with PLA, poly (alkyl cyanoacrylate) (PACA), and chitosan. Surface modification using PEG and mPEG could enable PEI nanoparticles to escape uptake by the mononuclear phagocytic system and reduce their cytotoxicity (Alvarez et al., 2014; Liu et al., 2015). In our study, FP21-mediated PEG-PEI complexes (FP21-PEG-PEI) were prepared using an evaporation method, loaded with growth-regulated oncogene alpha (GRO-α) short hairpin RNA (shRNA) plasmid as the model nanoparticle drug.

GRO-α is a cancer gene that plays an important role in the process of proliferation and malignant transformation of tumor cells (Casanova et al., 2002; Parkunan et al., 2016). The chemokine CXCL1 (GRO-α encoding protein) is the key component of tumor cells in a wide range of normal and pathological conditions, including inflammation, angiogenesis, wound healing, and tumor invasion (Casanova et al., 2002; Boro and Singh, 2016; Mao et al., 2017). The GRO-α gene is highly expressed in ovarian cancer tissue and cells and is strongly linked with ovarian cancer cell proliferation, invasion, and metastasis; 83% of human ovarian carcinoma cells express the GRO-α gene (Fimmel et al., 2007). We designed and synthesized four sequences of GRO-α shRNA using BLOCK-iT RNAi Designer software (Invitrogen, Carlsbad, CA), and then screened out one sequence that had the highest downregulated efficiency of the four. To verify the drug delivery system, we selected the GRO-α shRNA plasmid as a model drug and investigated the efficacy of the targeted complex *in vitro* and *vivo*.

In this study, we conjugated FP21 peptide with PEG-modified PEI to form a safe and efficient nonviral gene delivery carrier. We determined that D-FP21 is a more efficient ligand *in vivo* and *in vitro* for ovarian tumor cells compared with L-FP21. The D-FP21-PEG-PEI/pGRO-α complexes were a more efficient drug *in vivo* for ovarian tumor treatment compared with L-FP21-PP/pGRO-α complexes. To the best of our knowledge, the application of D-FP21-PP/pDNA in a nonviral gene delivery system has not previously been reported.

## Materials and methods

### Materials

PEI (branched, *M_w_* = 25000), mPEG maleimide (mPEG-Mal, *M_w_* = 3500), and maleimide PEG N-succinimidyl ester (Mal-PEG-NHS, *M_w_* = 3500) were purchased from Kaijian Technology Co., Ltd. (Beijing, China).

L-FP21, D-FP21, Rhodamine-L-FP21, and Rhodamine-D-FP21 were purchased from ChinaPeptides (Shanghai, China) (Figure S3). Rat serum was obtained from Jianlun Biology Technology Co. (Guangzhou, China). Aminopeptidase M (leucine aminopeptidase, microsomal from porcine kidney Type VI − S, lyophilized powder, EC 3.4.11.2) and the Endotoxin-free Plasmid Maxiprep Kit used to purify the plasmid DNA before application were obtained from Sigma–Aldrich (USA). Cell Counting Kit-8 (CCK-8 Kit) was purchased from Beyotime Biotechnology (Tianjing, China). Odyssey Blocking Buffer was obtained from LI-COR Biosciences (Lincoln, Nebraska, USA). The plasmids encoding enhanced green fluorescence protein (EGFP; pEGFP-N) were purchased from KeyGEN BioTECH (Nanjing, China). The vector construction of GRO-α shRNA was pcDNA6.2-GW/Emgfp-miR (Invitrogen, Carlsbad, CA), and the target knockdown gene encoding was TGGTTTT GGCCACTG. The antibiotic used to apply selective pressure, spectinomycin dihydrochloride, was also obtained from Sigma–Aldrich.

### Cell culture and animals

The HO8910 ovarian cancer cell line and HEK 293 T human embryonic kidney cell line were purchased from the Shanghai Institute of Cell Biology and were cultured in Dulbecco’s modified Eagle’s medium (DMEM) (Invitrogen, Beijing, China) containing 10% fetal bovine serum (FBS) (Gibco), 100 U/mL penicillin, and 100 mg/mL streptomycin (Gibco). Female BALB/c nude mice (18 g) were purchased from Shanghai SLAC Laboratory Animal Co., Ltd. (Shanghai, China) and were kept under specific-pathogen-free conditions. The animal experiments were evaluated and approved by the Ethics Committee of Fudan University. In addition, all methods in this study were performed in accordance with the manufacturers’ protocols and consolidated standards of reporting trials (CONSORT) standards.

### *In vitro* stability studies of peptides

The L-FP21 and D-FP21 peptides were diluted to 0.5 mg/mL and incubated at 37 °C for 12 h for the biostability test against 20% rat serum. At predetermined time intervals, 100 μL of the sample was removed and analyzed using reverse-phase high-performance liquid chromatography (HPLC; Waters Acquity H-Class Bio, Waters Co. Ltd., Milford, MA) on a bridged ethylene hybrid (BEH) C18 column (2.1 mm ×50 mm, 1.7 μm) using a gradient method. The mobile phase A was purified water containing 0.1% (v/v) formic acid (FA). The mobile phase B was acetonitrile containing 0.1% FA. The 30-min linear elution gradient ran from 5% B to 45% B in 5 min at a flow rate of 0.6 mL/min with a detection wavelength of 214 nm. We used aminopeptidase to further investigate the stability of L-FP21 or D-FP21 in proteolytic enzymes. The aminopeptidase was added with a final concentration of 1 μg/mL. The peptides were then analyzed as described above. All the experiments were performed in triplicate.

#### Preparation of L-FP21-PEG-PEI and D-FP21-PEG-PEI

In a typical procedure, D-FSH (4 equiv.) and Mal-PEG-NHS (1 equiv.) were dissolved in dimethylformamide. After 4 h under stirring, the reaction was terminated by adding deionized water to the mixture. Then, the solution was transferred to an Amicon Ultra-4 tube (3 kDa) and centrifuged under 3000 rpm for 45 min. The residual solution was diluted with deionized water, and the above procedure was repeated 10 times. The purified product was lyophilized and denoted D-FP21-PEG-Mal.

D-FP21-PEG-PEI was synthesized by the reaction of D-FP21-PEG-Mal and PEI. First, 200 mg of PEI was dissolved in 100 mL of deionized water, and the pH of the solution was then adjusted to 7.4 using 1 N HCl. Next, 240 mg of D-FP21-PEG-Mal was added to the solution of PEI, and the mixture was stirred for 24 h at room temperature. The crude D-FP21-PEG-PEI was transferred to an Amicon Ultra-4 tube (10 kDa) and centrifuged at 800 × g for 45 min. The residual solution was diluted with deionized water, and the above procedure was repeated six times. The purified product was lyophilized and denoted D-FP21-PEG-PEI. The mPEG-PEI was obtained as described above, and the preparation of L-FP21-PEG-PEI was the same as that of D-FP21-PEG-PEI.

#### Preparation of polymer/pDNA complexes

The obtained polymers were dissolved in phosphate buffered saline (PBS), and then equal volumes of pDNA solution were added dropwise. The final concentration of pDNA was 500 μg/mL. After vortexing for 30 s, the polymer/pDNA complexes were incubated at room temperature for 30 min. The polymer–nitrogen/pDNA-phosphorus ratios (N/P ratios) were determined using the following equation: *N/P* = 7.53 × *m_PEI_/m_pDNA_*, where m_PEI_ and m_pDNA_ represent the mass of PEI and pDNA, respectively.

#### Cellular uptake

HO8910 ovarian cancer cells and 293 T cells were seeded in 48-well plates at a density of 4 × 10^4^ cells/well in 0.5 mL of DMEM with 10% FBS and incubated overnight at 37 °C. Rhodamine-L-FP21 and Rhodamine-D-FP21 at a final concentration of 5 μM were added to the plate and incubated for 4 and 12 h, respectively. The HO8910 and 293 T cells were washed with PBS (0.01 M, pH 7.4) twice and fixed with 4% formaldehyde for 10 min. The cells were washed with PBS twice, and the nucleus was dyed with 4′,6-diamidino-2-phenylindole for 15 min and subjected to fluorescence microscopy analysis. The 293 T cell line, a human embryonic kidney cell line, was used as a control group because it does not express *FSHR*. Sections were analyzed using a fluorescent microscope (Nikon Ti-E, Japan) with 350 nm (blue) and 515–560 nm (red) excitation filters.

#### Characterization

^1^H NMR spectra of D-FP21-PEG-PEI, L-FP21-PEG-PEI, and m-PEG-PEI were recorded on a Bruker Avance 400 spectrometer using D_2_O as the solvent; trimethylsilane was used as an internal standard. The hydrodynamic diameter and zeta potential were characterized using dynamic light scattering measurements (Zetasizer Nano-ZS90, Malvern, UK). Each sample was measured in triplicate.

#### Cytotoxicity assay

HO8910 cells were seeded in 96-well plates (5000 cells/well) and incubated for 24 h at 37 °C in 200 μL of DMEM containing 10% FBS. The PEI/pGRO-α, mPEI-PEG/pGRO-α, D-FP21-PP/pGRO-α, and L-FP21-PP/pGRO-α were prepared freshly with different N/P ratios (N/P = 4, 8, 12, and 16). Then, 0.9 μg of pDNA was added to each well and incubated with serum-free DMEM for 24 h. Next, 10 μL of the CCK-8 solution was added to each well of the plate, and the plate was incubated for 3.5 h in the incubator. The absorbance at 450 nm was measured using a Multiskan MK3 microplate reader (Thermo Fischer Scientific, Waltham, MA). The measurements for each sample were conducted in triplicate. The cellular toxicities of the GRO-α shRNA-loaded nanocomposite were measured using real-time cellular analysis (RTCA). The samples of PEI/pGRO-α, mPEI-PEG/pGRO-α, D-FP21-PP/pGRO-α, and L-FP21-PP/pGRO-α (N/P = 12) were diluted using a serum-free medium to a series of doses based on pGRO-α shRNA concentrations ranging from 2.2 to 143.3 μg/mL. The half maximal inhibitory concentration (IC_50_ value) was calculated based on the RCTA results.

#### *In vitro* gene transfection efficiency

HO8910 cells were seeded in 48-well plates at a density of 5 × 10^4^ cells per well and incubated overnight at 37 °C. Then, 20 μL of polymer/pEGFP-N complexes containing 1.2 μg of pEGFP-N at N/P ratios of 4, 8, 12, and 16 were added to each well. After incubation at 37 °C for 24 h, the HO8910 cells were lysed and suspended in PBS before being subjected to flow cytometry (FACS Calibur, BD Biosciences, San Jose, CA).

### *In vitro* anti-proliferation assay

The plasmid GRO-α shRNA that we designed is able to present transient inhibition expression of GRO-α by a vector expressing shRNA. HO8910 cells were seeded in 24-well plates at a density of 1 × 10^5^ cells per well in 0.8 mL of DMEM with 10% FBS and incubated overnight at 37 °C. Then, 50 μL of D-FP21-PP/pGRO-α, L-FP21-PP/pGRO-α, and mPEI-PEG/pGRO-α complexes (N/P = 12) containing 3 μg of pGRO-α shRNA were added to each well. Wells with 3 μg of pEGFP-N added were used as control groups. After incubation at 37 °C for 12 h, the HO8910 cells were lysed and seeded in 96-well plates. Then, the cell growth curves were automatically recorded on the xCELLigence RTCA DP System (Roche Applied Sciences) in real time (Pan et al., 2013; Roshan Moniri et al., 2015). The assays were performed in triplicate. The data were analyzed using the RTCA Software 1.2 program (Roche Diagnostics). All the data are presented as the mean normalized cellular index ± the standard error of the mean over time.

#### In-cell Western blot assays

The HO8910 cells were seeded in 96-well plates (3 × 10^4^ cells/well) and incubated overnight at 37 °C in 200 μL of DMEM containing 10% FBS. Samples of D-FP21-PP/pGRO-α, L-FP21-PP/pGRO-α, and mPEG-PEI/pGRO-α containing 0.9 μg of pGRO-α shRNA were added to the plate. Each sample was added in triplicate. After being incubated for 48 h, the samples were fixed using formaldehyde for 10 min, washed with 0.1% Triton X-100 in PBS five times, and blocked by 150 μL of LI-COR Odyssey Blocking Buffer for 90 min at room temperature. The cells were then incubated with rat anti-GRO-α antibody (1:150) and rabbit anti-actin antibody (1:150) in PBS for 2.5 h. This process was followed by three rinses in phosphate buffered saline Tween-20 and incubation with IRDye 700DX (red florescence)-conjugated goat anti-mouse florescence secondary antibody (1:500) and IRDye 800DX (green fluorescence)-conjugated goat anti-rabbit florescence secondary antibody (1:500) in the dark. The signal was detected semiquantitatively using the Odyssey Infrared Imaging System (Egorina et al., 2006; Lammi et al., 2015; Sakamoto and Liamptong, 2015) (LI-COR Biosciences Ltd.). For the statistical analyses, integrated intensities of the fluorescence in each well were calculated using LI-COR software. The expression level of the corresponding molecules was calculated as the ratio of the intensity of GRO-α proteins to actin.

#### *In vivo* evaluation of antitumor activity

The *in vivo* antitumor activities of D-FP21-PP/pGRO-α and L-FP21-PP/pGRO-α were evaluated using female BALB/c nude mice. Four- to five-week-old mice were inoculated with 7 × 10^6^ cells subcutaneously on the flank to establish xenograft tumors. Seven days after tumor implantation, the tumor volume reached approximately 150 mm^3^, and the mice were randomly divided into four treatment groups (7 mice per group): PBS, D-FP21-PP/pGRO-α, L-FP21-PP/pGRO-α, and mPEG-PEI/pGRO-α. They were intravenously injected separately with freshly prepared PBS, D-FP21-PP/pGRO-α, L-FP21-PP/pGRO-α, and mPEG-PEI/pGRO-α (containing 3.5 mg of pDNA/kg in 200 μL of PBS, N/P = 12) or PBS solution via the tail vein. This process was repeated six times on days 8, 11, 14, 17, 20, and 23 after implantation. The tumor volume was calculated using the formula: tumor volume (mm^3^) = (length × width^2^) × π/6. Additionally, the body weight of the mice in each group was also monitored for 30 days. To assess the drug toxicity to these major organs, two mice from each group (PBS, D-FP21-PP/pGRO-α, and mPEG-PEI/pGRO-α) were randomly selected from the nude mice and sacrificed, and the heart, liver, spleen, lung, and kidney were dissected and prepared for hematoxylin and eosin (H&E) staining. All the data are presented as mean ± standard errors. An independent *t*-test using SPSS 16.0 for Windows was used for two-group comparisons. *p < .05 and **p < .001 were used to signify statistical significance in this study.

## Results

### *In vitro* stability studies of peptides

**Figure 1.**
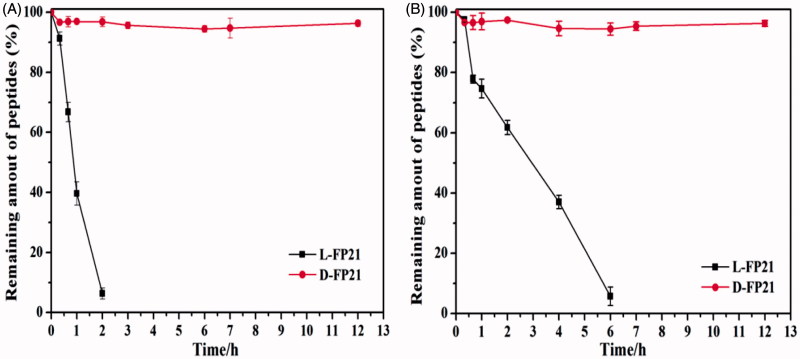
Stability of peptides in rat serum (A) and aminopeptidase M (B).

The results are presented in Figure 1. Compared with D-FP21, L-FP21 was degraded rapidly. It was completely degraded within 2 h in rat serum and 6 h in aminopeptidase. In contrast, the retro-inverso D-FP21 was much more stable, with no degradation even after 12-h incubation whether with rat serum or aminopeptidase. These findings suggest that D-peptides exhibit better biostability against proteolytic enzymes than their L-counterparts.

#### Synthesis and characterization of polymers

mPEG-PEI and D-FP21-PEG-PEI were synthesized as indicated in Figure S1 and the ^1^H nuclear magnetic resonance (NMR) spectroscopy results are presented in Figure S2. The results were as follows: 3.6–3.7 ppm (m, -OCH2CH2) for PEG, 2.4–3.0 ppm (m, -CH2CH2NH-) for PEI, and 6.6–7.0 ppm for D-FP21. The substituted degree of PEG was 4.8 for mPEG-PEI and 5.5 for D-FP21-PEG-PEI, which was close to the theoretical value of 5.0. These details indicate that D-FP21 was successfully linked to the polymer.

### Characterization of polymer/pDNA complexes

The particle sizes and zeta potentials of polymer/pGRO-α shRNA complexes are important properties that can affect the transfection efficiency in a cell. As shown in Table S1, the sizes of the complexes decreased with the increasing N/P ratio, whereas the zeta potentials increased. At the same N/P ratio, the D-FP21-PEG-PEI/pGRO-α and L-FP21-PEG-PEI/pGRO-α complexes were similar in terms of sizes and zeta potentials. The use of D-amino acids did not affect the characteristics of the polymers. At N/P = 12, the particle sizes for PEI, mPEG-PEI/pGRO-α, L-FP21-PP/pGRO-α, and D-FP21-PP/pGRO-α were 561.1 ± 43.5 nm, 217.8 ± 5.3 nm, 157.4 ± 4.1 nm, and 168.9 ± 5.1 nm, respectively, and their zeta potentials were 29.75 ± 0.85 mV, 11.78 ± 0.36 mV, 6.94 ± 0.49 mV, and 5.19 ± 0.78 mV, respectively.

### Cellular uptake

**Figure 2.**
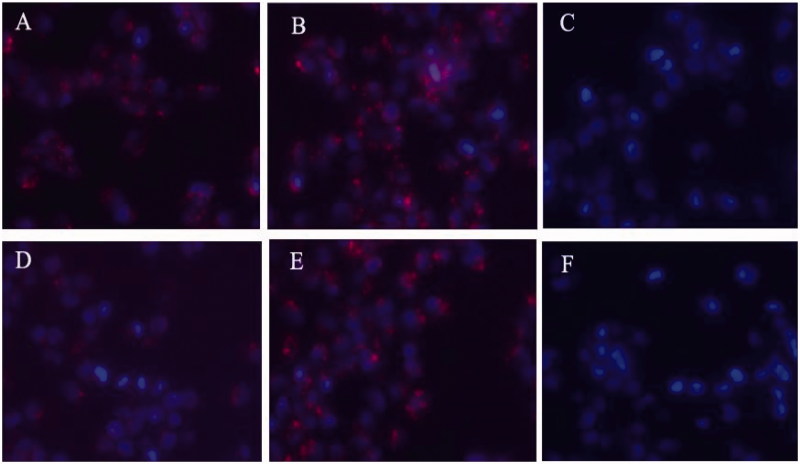
Uptake of Rhodamine-L-FP21, and Rhodamine-D-FP21 by HO8910 cells and 293T cell. Images A,B are fluorescence images of uptake of Rhodamine-L-FP21, and Rhodamine-D-FP21 by HO8910 cells after 4-h incubation, respectively. Images D,E are fluorescence images of uptake of Rhodamine-L-FP21, and Rhodamine-D-FP21 by HO8910 cells after 12-h incubation, respectively. Images C,F are fluorescence images of uptake of Rhodamine-L-FP21 and Rhodamine-D-FP21 by 293T cells after 4-h incubation, respectively: red represents red fluorescence protein expressed by Rhodamine; DAPI represents cell nuclei stained with DAPI. Original magnification, 200×.

The fluorescence images of cellular uptake are presented in Figure 2. After 4-h incubation, the HO8910 cells exhibited good uptake for both of rhodamine-D-FP21 and rhodamine-L-FP21 polypeptides. However, after 12-h incubation, the FI of the Rhodamine-L-FP21 group was clearly attenuated; in contrast, that of the Rhodamine-D-FP21 group showed no apparent changes. The 293 T cells which are FSHR expression negative cells showed no uptake of the two peptides at 4 or 12 h.

### Cytotoxicity assay

**Figure 3.**
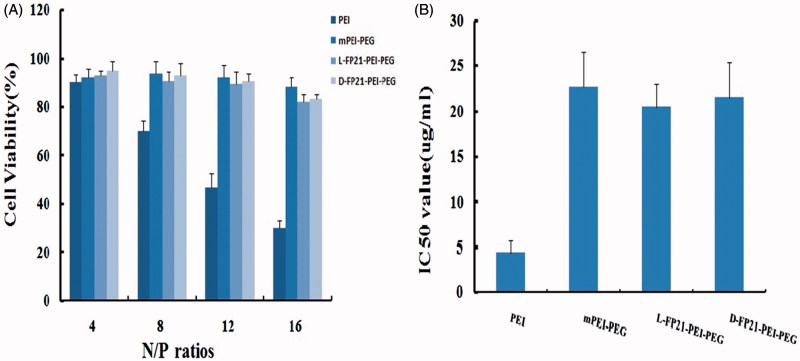
Cell viabilities (A) and IC_50_ value (B) of mPEG-PEI/ pGRO-α shRNA and L,D-FP21-PEG-PEI/ pGRO-α shRNA compared with PEI/ pGRO-α shRNA in HO8910 cells.

We used the CCK-8 assay and RTCA to evaluate the cytotoxicity of PEI derivative/pGRO-α complexes. Figure 3(A) indicates that the PEI/pGRO-α complex had a greater cytotoxic effect than the PEGylated polymer/pGRO-α complexes. At N/P ratios from 8 to 16, the cell viability decreased from 70.1% to 30.2%, which was much lower than those of mPP/pGRO-α and L, D-FP21-PP/pGRO-α treated cells, which exhibited average cell viabilities >82%. RTCA showed that the IC_50_ of PEI/pGRO-α, mPP/pGRO-α, L-FP21-PP/pGRO-α, and D-FP21-PP/pGRO was 4.5 μg/mL, 22.3 μg/mL, 23.4 μg/mL, and 21.6 μg/mL, respectively (Figure 3(B)). In addition, no difference was observed in the cell viabilities following D-FP21-PP/pGRO-α and L-FP21-PP/pGRO-α treatment at any N/P rate. The D-amino acids did not increase the cytotoxicity of the drug delivery systems.

### *In vitro* gene transfection efficiency

**Figure 4.**
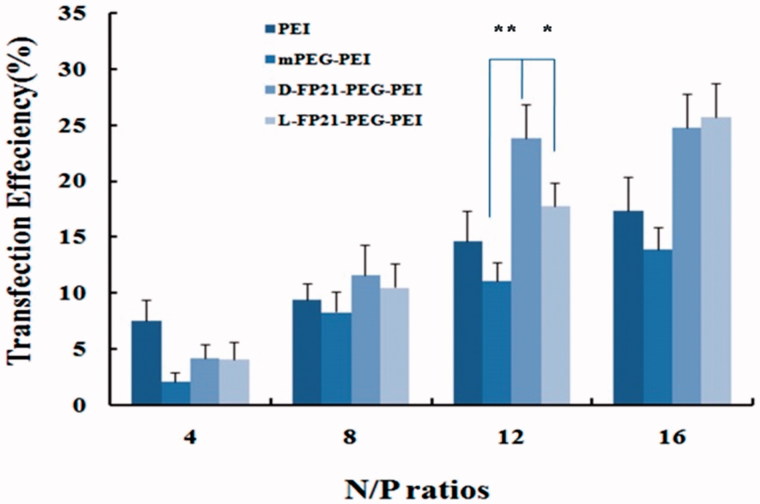
*In vitro* transfection efficiency of HO8910 cells. Luciferase expression levels of mPEG-PEI/ pEGFP-N, L, D-FP21-PEG-PEI/ pEGFP-N, and PEI/ pEGFP-N at different N/P ratios in transfected cells. *indicates *p* < .05, **indicates *p* < .001.

EGFP expression of HO8910 cells was detected and analyzed with a quantitative luciferase assay using flow cytometry, and the results are presented in Figure 4. The transfection efficiency of all PEGylation complex/pEGFP-N increased steadily, with the N/P ratio increasing. At N/P ratios of 12 and 16, the EGFP expression rate of L, D-FP21-PP/pEGFP-N was much higher than that of mPP/pEGFP-N and PEI/pEGFP-N. It is reasonable to assume that the 21-amino acid polypeptides had strong targeting specificity, which clearly facilitated the increase in transfection efficiency. At an N/P ratio of 12, gene transfection efficiency of the D-FP21-PP/pEGFP-N, L-FP21-PP/pEGFP-N, and mPP/pEGFP-N complexes were 11.1 ± 2.7%, 23.8 ± 3.1%, and 17.8 ± 3.0%, respectively. The D-FP21-PP/pEGFP-N complex was 1.34- and 1.95-times more efficient than L-FP21-PP/pEGFP-N and mPP/pEGFP-N, respectively. However, the transfection efficiency of the PEGylation polymers was higher at an N/P ratio of 16, the cell cytotoxicity increased. Based on the cytotoxicity assay and gene transfection efficiency results, we selected an N/P ratio of 12 for PEGylation polymers for our remaining experiments.

### *In vitro* anti-proliferation assay

**Figure 5.**
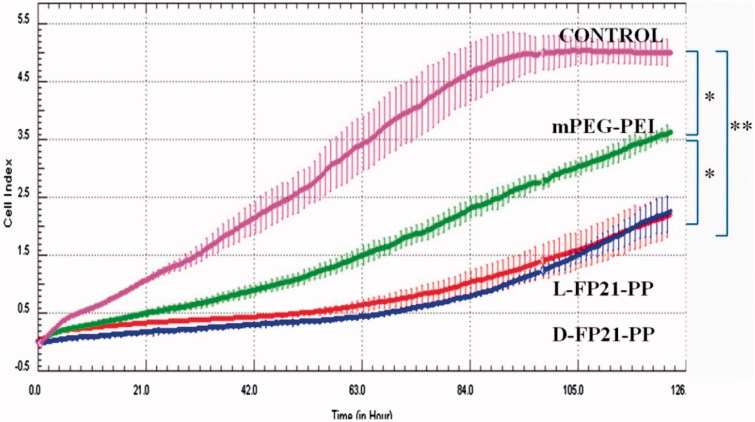
*In vitro* proliferation assay. *Vitro* anti-proliferation assay of PBS and PEGylation polymers/pGRO-α by RTCA (CONTROL, mPEG-PEI, L-FP21-PP, D-FP21-PP represent the groups treated by PBS, mPEG-PEI/ pGRO-α complex, L-FP21-PEG-PEI/ pGRO-α complex and L-FP21-PEG-PEI/ pGRO-α complex; *indicates *p* < .05, **indicates *p* < .001).

We investigated whether the PEGylation polymers/pGRO-α could inhibit tumor cell proliferation *in vitro*. Figure 5 shows that cell proliferation in the PEGylation polymers/pGRO-α groups was significantly inhibited compared with that of the control group (*p* < .01); In addition, the mPP/pGRO-α treated group was intermediate. No differences were observed between the D-FP21-PP/pGRO-α treated group and L-FP21-PP/pGRO-α treated group.

#### In-cell Western blot assays

The In-Cell Western (ICW) assay has been applied for semiquantitative analysis of cellular GRO-α protein to assess the effect of the nanodrugs. As shown in Figure S4, the GRO-α protein levels of the L, D-FP21-PP/pGRO-α treated group significantly decreased compared with those in the control group (*p* < .001). Similar changes were observed in the mPP/pGRO-α treated group (*p* < .05). The difference in the GRO-α protein level between the D-FP21-PP/pGRO-α treated group and the L-FP21-PP/pGRO-α treated group was not statistically significant. These results indicate that the significant increase in the transfection efficiency of L, D-FP21-PP in the HO8910 cells relative to the non-targeted polymer can be attributed to receptor-mediated endocytosis enhancing the capacity of the FP21-PP/pGRO-α complex to enter cancer cells, thus facilitating its anti-proliferative effect.

#### *In vivo* evaluation of antitumor activity

**Figure 6.**
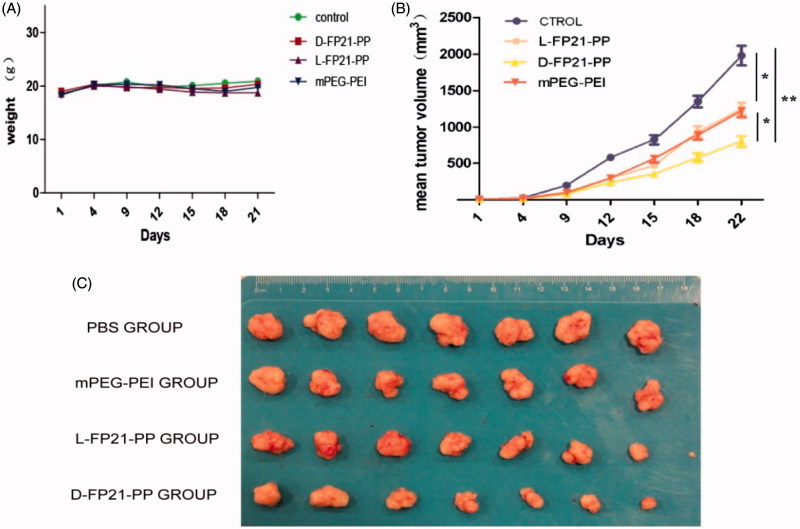
A: The change of weight of nude mice treated by PEG-PEI nanoparticles drug. B: Tumor volumes in PBS, mPEG-PEI/ pGRO-α and L, D-FP21-PEG-PEI/ pGRO-α groups (*n* = 7) (*indicates *p* < .05, **indicates *p* < .001). C: Tumors stripped from nude mice treated by PBS, mPEG-PEI/ pGRO-α and L, D-FP21-PEG-PEI/ pGRO-α on day 30.

We used plasmid GRO-α shRNA as a therapeutic gene packed in PEGylation polymers to form a targeted gene delivery system for the treatment of ovarian cancer. In day 25 of observation, the growth of tumor spheroids was clearly inhibited by D-FP21-PP/pGRO-α, L-FP21-PP/pGRO-α, and mPP/pGRO-α compared with the PBS control group (Figure 6(B)). A significant reduction in tumor growth occurred for the D-FP21-PP/pGRO-α treated group (*p* < .001). The reduction in tumor growth of the L-FP21-PP/pGRO-α and mPP/pGRO-α treated groups showed no statistically significant differences as compared with each other. At day 25, the tumor spheroid volumes of the D-FP21-PP/pGRO-α, L-FP21-PP/pGRO-α, and mPP/pGRO-α treated groups were 58.5%, 33.3%, and 26.3% of the control group volume, respectively (Figure 6(C)). No significant body weight changes were observed (Figure 6(A)). Besides slight necrosis with inflammatory cell infiltration in the liver tissue in the groups treated with D-FP21-PP/pGRO-α and mPP/pGRO-α, there were no apparent histopathological abnormalities or lesions in the heart, spleen, lung, or kidney tissues observed for any of the treatment groups (Figure S5). These results demonstrate that tumor inhibition by pGRO-α shRNA nanoplexes occurs with an effective and safe delivery system characterized by high specificity and affinity of polypeptide ligand targeting of the tumor cells.

## Discussion

Receptor expression is a key requirement for successful documentation of ligand-mediated delivery of polymer/DNA complexes to cancer cells. Previously, we found that 17 of 24 primary human ovarian carcinoma specimens (70.83%) expressed FSHR, and there was no FSHR expression in the main organs of BALB/c mice bearing human ovarian carcinoma, such as the heart, liver, spleen, lung, and kidney (Zhang et al., 2009). In the present study, we initially examined FSHR expression in five human ovarian cancer cell lines. The expression of the GRO-α gene in the cell lines was also investigated. Compared with the A2780, HEY, and SKOV3 cell lines, the HO8910 cell line had high expression of both FSHR and GRO-α, as shown by western blotting (Figure S6). Accordingly, we selected HO8910 cells as the *in vitro* and *in vivo* ovarian cancer cellular models.

In this study, we developed a novel targeted gene therapy delivery system for ovarian cancer. The nanoparticles conjugated to FP21 can deliver gene drugs to *FSHR*-expressing cells. Previously, we had demonstrated that FP21 can bind to the FSHR of ovarian cancer cells specifically and did not affect cancer cell proliferation and metastasis.

The stability of the targeting ligand is important. L-peptides (L-FP21), which we used previously, are linear structures and can be easily degraded by plasma proteases. D-peptides (D-FP21), which we synthesized in the present study, have very similar side chains to the original structure and have the same targeting property and improved biological stability in rat serum or aminopeptidase. The cellular uptake assay results indicated that the two polypeptides can specifically recognize HO8910 cells rather than FSHR expression negative cells, and that D-FP21 has a better uptake rate for HO8910 cells than the parental L-FP21 in the 12-h incubation test. However, the D-FP21-PP/pDNA complexes did not demonstrate superiority in the *in vitro* assay. We presume that the density of drug added to the panel is relatively high. The drug concentration in the panel was saturated. Predominantly, large amounts of drug were taken up through specific cell-mediated endocytosis in a short time before degradation. Thus, the anti-proliferative effects and gene transfection efficiency of the D-FP21-PP/pDNA complexes showed equipotency to that of the L-FP21-PP/pDNA complexes in the *in vitro* assay. Nevertheless, in the *in vivo* anti-tumor study, the nanodrug modified by the D-peptides demonstrated superiority. Both drugs were injected into the blood circulation, and the D-FP21-PP/pDNA complexes had higher anti-tumor activity. The retro-inverso peptide ligand modification of the nanodrug enhanced the anti-tumor effect significantly by reducing the tumor spheroid volumes of tumor-bearing nude mice from 33.3% to 58.5%. This is likely due to the resistance of the ligand to hydrolysis catalyzed by endogenous peptidases in the blood, which results in relatively better biostability and tumor targeting. This success will inspire more in-depth research in this emerging field.

The positive charge on the surface of PEI facilitates its high transfection efficiency, and simultaneously increases its cytotoxicity. The PEI/pGRO-α complexes exhibited large sizes and high zeta potentials. PEGylation can block a part of the positive charge of PEI, reducing PEI cytotoxicity, and decreasing aggregation. This was the reason the PEGylated polymer/pGRO-α complexes we tested were smaller and had lower zeta potentials than the PEI/pGRO-α complexes. The IC_50_ of the PEGylated polymer/pGRO-α complexes was four times higher than that of the PEI derivative/pGRO-α complexes. The *in vivo* anti-tumor activity study showing fewer histopathological abnormalities or lesions in the nude mice can be partly explained by the PEGylated polymer complexes being used to deliver drugs to the foci while minimizing cytotoxicity in unrelated normal organs.

## Conclusion

We previously established an ovarian cancer targeted delivery system utilizing FP21 polypeptide as the targeting ligand, and the results indicated that further development and optimization of FSH-peptide-mediated PEI for targeted therapy of ovarian cancer was warranted. In this study, we demonstrate that D-FP21-PP/pDNA complexes exhibit improved long-term stability *in vitro* and anti-tumor efficacy *in vivo* compared with L-FP21-PP/pDNA complexes. In conclusion, D-FP21-PEG-PEI/pDNA complex appears to be a promising gene delivery system with potential applications for ovarian cancer treatment.
